# Statistical Methods for Adjusting Estimates of Treatment Effectiveness for Patient Nonadherence in the Context of Time-to-Event Outcomes and Health Technology Assessment: A Systematic Review of Methodological Papers

**DOI:** 10.1177/0272989X19881654

**Published:** 2019-10-24

**Authors:** Abualbishr Alshreef, Nicholas Latimer, Paul Tappenden, Ruth Wong, Dyfrig Hughes, James Fotheringham, Simon Dixon

**Affiliations:** Health Economics and Decision Science, School of Health and Related Research (ScHARR), University of Sheffield, Sheffield, South Yorkshire, UK; Health Economics and Decision Science, School of Health and Related Research (ScHARR), University of Sheffield, Sheffield, South Yorkshire, UK; Health Economics and Decision Science, School of Health and Related Research (ScHARR), University of Sheffield, Sheffield, South Yorkshire, UK; Health Economics and Decision Science, School of Health and Related Research (ScHARR), University of Sheffield, Sheffield, South Yorkshire, UK; Centre for Health Economics & Medicines Evaluation (CHEME), Bangor University, Bangor, Gwynedd, UK; Sheffield Kidney Institute, Sheffield Teaching Hospitals NHS Trust, Sheffield, South Yorkshire, UK; Health Economics and Decision Science, School of Health and Related Research (ScHARR), University of Sheffield, Sheffield, South Yorkshire, UK

**Keywords:** causal inference, cost-effectiveness analysis, medication nonadherence, noncompliance, survival analysis

## Abstract

**Introduction.** Medication nonadherence can have a significant negative impact on treatment effectiveness. Standard intention-to-treat analyses conducted alongside clinical trials do not make adjustments for nonadherence. Several methods have been developed that attempt to estimate what treatment effectiveness would have been in the absence of nonadherence. However, health technology assessment (HTA) needs to consider effectiveness under real-world conditions, where nonadherence levels typically differ from those observed in trials. With this analytical requirement in mind, we conducted a review to identify methods for adjusting estimates of treatment effectiveness in the presence of patient nonadherence to assess their suitability for use in HTA. **Methods.** A “Comprehensive Pearl Growing” technique, with citation searching and reference checking, was applied across 7 electronic databases to identify methodological papers for adjusting time-to-event outcomes for nonadherence using individual patient data. A narrative synthesis of identified methods was conducted. Methods were assessed in terms of their ability to reestimate effectiveness based on alternative, suboptimal adherence levels. **Results.** Twenty relevant methodological papers covering 12 methods and 8 extensions to those methods were identified. Methods are broadly classified into 4 groups: 1) simple methods, 2) principal stratification methods, 3) generalized methods (g-methods), and 4) pharmacometrics-based methods using pharmacokinetics and pharmacodynamics (PKPD) analysis. Each method makes specific assumptions and has associated limitations. Five of the 12 methods are capable of adjusting for real-world nonadherence, with only g-methods and PKPD considered appropriate for HTA. **Conclusion.** A range of statistical methods is available for adjusting estimates of treatment effectiveness for nonadherence, but most are not suitable for use in HTA. G-methods and PKPD appear to be more appropriate to estimate effectiveness in the presence of real-world adherence.

Patient nonadherence to medications can have a significant negative impact on treatment effectiveness and health care costs and has the potential to alter the conclusions of economic evaluations and health technology assessments (HTAs).^1–3^ An economic evaluation typically assesses the cost-effectiveness of a new treatment compared to standard treatment using evidence on clinical effectiveness and costs. Intention-to-treat (ITT) analysis, which compares randomized groups regardless of nonadherence or withdrawal, is a well-established method for estimating treatment effectiveness from randomized controlled trials (RCTs).^4^ However, ITT estimates may not be relevant if the HTA aims to assess the effectiveness of treatment given real-world adherence patterns.^5,6^

There is evidence to show that adherence in the real world is likely to differ from RCTs (depending on the type of treatment, disease area, and health care setting), which leads to uncertainty around the actual effectiveness of treatments.^7–9^ Clinical effectiveness estimates have a direct impact on cost-effectiveness; consequently, a cost-effectiveness analysis (CEA) that does not incorporate nonadherence may produce misleading conclusions regarding the value of the technology. In the HTA context, we are interested in effectiveness estimates inferred to the entire study population (as defined by scope and study eligibility criteria), which can be identified at baseline, as opposed to estimates focused on a latent subgroup of the population (e.g., compliers). Moreover, HTA agencies are interested in adjustment methods, which can be used for reestimating treatment effectiveness for any given level of adherence, to reflect potential real-world adherence levels.^10,11^

The fundamental issue in estimating effectiveness associated with alternative adherence levels is the methodological challenge associated with adjusting for time-dependent confounding. In this context, time-dependent confounders are prognostic factors that predict subsequent nonadherence and outcomes, yet are themselves predicted by previous nonadherence.^12^ When time-dependent confounders are present, more complex methods than simple regression adjustment are needed because simple regression adjustment is unable to deal with variables that predict adherence and are also an intermediate step between adherence and outcome. A range of methods has been proposed for estimating the causal effect of treatments in the presence of nonadherence, but little guidance exists about their relative advantages,^13–15^ and not all deal with time-dependent confounding appropriately. In addition, these methods have been designed, principally, to reestimate effectiveness assuming perfect adherence, whereas HTA requires reestimation for suboptimal (real-world) adherence.

The aims of the review are to systematically identify approaches for adjusting for nonadherence in the context of time-to-event outcomes using individual patient data in RCTs, to describe how each is undertaken, and to assess their suitability for reestimating effectiveness based on alternative, suboptimal adherence levels.

## Methods

### Review Question and Protocol

The review question was as follows: “What methods have been proposed in the methodological literature to account for the impact of nonadherence to treatments on clinical effectiveness and cost-effectiveness?” The review approach adheres to published international guidelines for undertaking and reporting systematic reviews, and methods were prespecified in a protocol.^16–20^

### Search Strategy

A “Comprehensive Pearl Growing” (CPG) technique^17^ and 2-stage iterative search approach was used across 7 databases (MEDLINE, Embase, Cochrane Library, EconLit, Scopus, Web of Science, MathSciNet). Databases were searched for potentially relevant papers published in English from inception to February 9, 2018 (first stage search), to May 23, 2018 (second stage search). The database searches were complemented by citation searches and reference list checking for each “pearl” (key paper) to identify additional relevant papers. The search approach was designed to identify the initial paper proposing the method (or articles reporting extensions to a previously developed method), rather than articles reporting the application of methods in studies.

The database search strategy comprises keywords for patient adherence combined with methods terms and focused MeSH headings of known pearls. The second stage search was informed from the collective analysis of newly identified pearls title, abstract, keywords, and MeSH and floating headings using the online Yale MeSH Analyzer Tool.^13,21–28^ Search terms and strategies are provided in online Supplementary Appendix A.

### Inclusion and Exclusion Criteria

The selection of papers included for narrative synthesis was conducted in 2 stages: 1) records retrieved from all sources were screened by titles followed by abstracts screening, and 2) potentially relevant full-text articles were assessed for eligibility using the inclusion and exclusion criteria (Suppl. Table S1 in Appendix B). One author (AA) screened all potentially relevant papers retrieved. A second author (SD) independently screened a subset of papers against the eligibility criteria. Disagreements between the 2 reviewers were resolved by discussion, and a consensus was reached on the final list of included papers. Expert opinion was obtained from 2 experts (DH, IW) for recommendation of additional papers.

### Data Extraction

A data extraction form was developed to extract the basic information and key characteristics for each method identified (Suppl. Table S2 in Appendix B).

### Data Synthesis

A narrative data synthesis approach was followed for each relevant method identified and its extensions. This included a description of the key characteristics of each method, as specified by the appraisal framework (Suppl. Table S3 in Appendix B).^29^ As part of this, we assessed which forms of nonadherence the method is capable of addressing, using the classification developed by Vrijens and colleagues.^28^ This classification differentiates between 3 stages of medication nonadherence: 1) initiation (when the first dose is taken by the patient), 2) implementation (how closely the actual dosage of a patient corresponds to the prescribed dosing regimen), and 3) persistence (time to discontinuation or end of therapy).^28^

We provide a brief description of the concept of each adjustment method, together with the causal model, its estimand (defined in the next section), key assumptions, and limitations. We assess whether the method is capable of reestimating effectiveness for other suboptimal levels of adherence (as opposed to optimal adherence). This assessment was based on the capability of the method to estimate the treatment effect under alternative counterfactual adherence levels (i.e., not observed adherence levels) given the adherence level and treatment effect actually observed in the trial. Finally, we assess the appropriateness of nonadherence adjustment methods for the HTA context based on criteria developed by the authors. The criteria were 1) the suitability of the estimand (as described in the next section), 2) the types of nonadherence the method is capable of dealing with, and 3) whether it is possible to use the method to account for real-world nonadherence levels.

### Possible Estimands and Suitability for HTA

An estimand is the parameter of interest estimated by the statistical method that we can use to make inferences about a population using a sample from that population.^5,30^ A range of possible estimands was identified, but only a few are appropriate for HTA. In the HTA context, resource allocation decisions are usually made for a specified population defined by the scope for each decision problem. Hence, the estimands of interest are those covering the entire study population (as specified by the RCT eligibility criteria), and this should be identifiable at baseline for resource allocation decision making. Therefore, estimands focused on latent subgroups of patients (e.g., compliers) may not be appropriate for the HTA context.

## Results

### Overview of Included Papers

**Figure 1:**
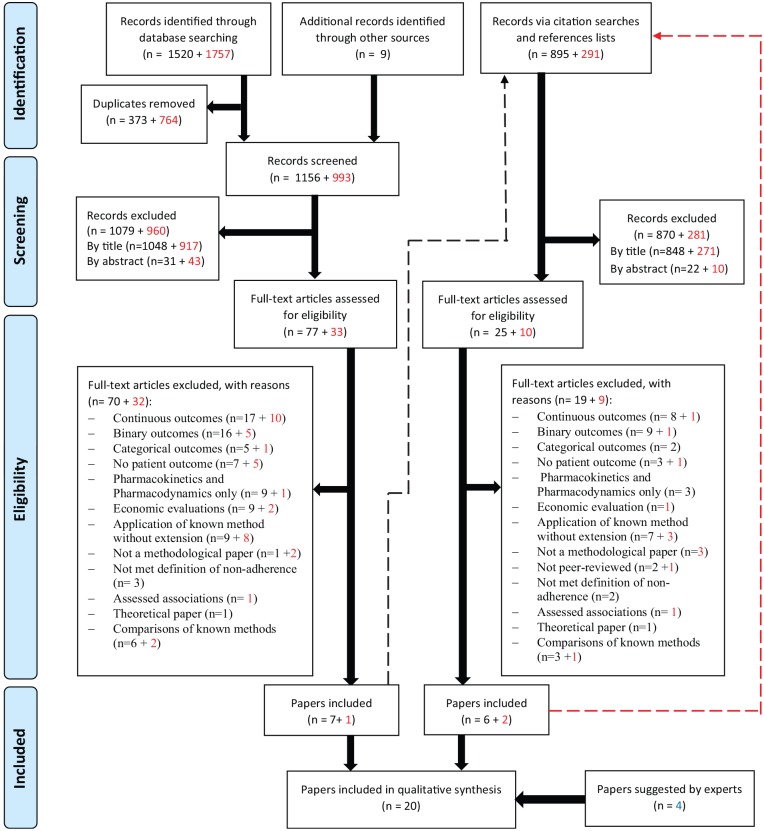
PRISMA Flow Diagram. PRISMA, preferred reporting items for systematic reviews and meta-analyses. Numbers in red represent records from the 2^nd^ stage of searches. The dashed lines show that citation searches and references lists checking were done for pearls identified from databases searching. Papers excluded for the reason of “comparison of known methods” are included in the citation searches and references lists checking as these were considered relevant for this purpose.

This review includes 20 papers describing 12 methods and 8 extensions to those methods.^22,31–49^ In total, the searches resulted in 4472 records (Figure 1). The included papers were published between 1992 and 2018 (inclusive); the majority were published in the *Statistics in Medicine* journal (30%) and *Biometrics* journal (25%). Other characteristics of included papers are given in Supplementary Table S4 in Appendix B.

### Taxonomy of Methods

A taxonomy of methods for adjusting estimates of treatment effectiveness for nonadherence in the context of time-to-event outcomes is proposed (Table 1). The purpose of the taxonomy is to increase understanding of the concept behind each method and its relation to other methods in terms of estimands and estimators.^30^ The structure of the taxonomy was initially developed by 1 author (AA) and further revised based on consultations with other authors (NL, PT, DH, JF, SD) and an expert in causal inference methods (IW).

**Table 1:** Taxonomy of Methods for Adjusting Treatment Effectiveness for Nonadherence in the Context of Time-to-Event Outcomes

Methods Group	Method Subcategory	Method/Extension	Reference
Simple methods	ITT^a^	Intention-to-treat (ITT) analysis	Yu et al., 2015^46^
	PP	Per-protocol (PP) analysis	Wu et al., 2015^45^
	AT	As-treated (AT) analysis	Korhonen et al., 1999^34^
Principal stratification methods	CPH with PLE	Cox proportional hazards (CPH) model with partial likelihood estimator (PLE)	Cuzick et al., 2007^41^
	MCC	Markov compliance class (MCC) model in a 3-stage method (3SM)	Lin et al., 2007^42^
	Wtd PP	Weighted per-protocol (Wtd PP) analysis using a proportional hazards model with an expectation-maximization (EM) estimator	Li and Gray, 2016^47^
	C-PROPHET	Compliers PROPortional Hazards Effect of Treatment (C-PROPHET)	Loeys and Goetghebeur, 2003^39^
	IV	Instrumental variable (IV) with likelihood estimator	Baker, 1998^32^
		IV extension: IV with plug-in nonparametric empirical maximum likelihood estimation (PNEMLE)	Nie et al., 2011^43^
		IV extension: transformation promotion time cure model with maximum likelihood estimation to estimate the complier average causal effect (CACE) and the complier effect on survival probability (CESP)	Gao and Zheng, 2017^48^
G-methods	MSMs	Marginal structural models (MSMs) with inverse probability of censoring weighting (IPCW)	Robins and Finkelstein, 2000^22^
		MSM extension: MSMs with inverse probability of treatment weighting (IPTW)	Hernan et al., 2001^35^
	SNFTMs	Structural nested failure time models (SNFTMs) with G-estimation	Robins et al., 1992^31^
	RPSFTMs	Rank-preserving structural failure time models (RPSFTMs) with G-estimation	Loeys et al., 2001^36^
		RPSFTM extension: incorporating covariates to improve the precision of estimators	Korhonen and Palmgren, 2002^37^
		RPSFTM extension: improving the efficiency of the estimators	Loeys and Goetghebeur, 2002^38^
		RPSFTM extension: allowing for dependent censoring	Matsui, 2004^40^
		RPSFTM extension: choice of model and impact of recensoring	White and Goetghebeur, 1998^33^
Pharmacometrics-based methods	PKPD	Pharmacokinetics and pharmacodynamics (PKPD)–based method	Pink et al., 2014^44^
		PKPD extension: modeling varying implementation and persistence types of nonadherence	Hill-McManus et al., 2018^49^

a. ITT does not adjust for nonadherence but is included in the taxonomy as a “do nothing” approach (i.e., ignoring nonadherence).

In the proposed taxonomy, methods are broadly classed into 4 groups: 1) simple methods that do not appropriately adjust for nonadherence; 2) principal stratification methods for estimating the complier average causal effect (CACE) estimand^50^; 3) generalized methods (g-methods), which are based on the counterfactual outcome framework originally developed by Neyman^51^ and Rubin^52^ for estimating the effect of time-fixed treatments, as well as further extended by Robins et al.^53,54^ for time-varying treatments; and 4) pharmacometric-based methods as a unique approach using pharmacokinetics and pharmacodynamics (PKPD) analysis commonly used in clinical trials for evaluating newly developed pharmacological interventions. The estimand and key assumptions used by each method are provided in Table 2, and the appropriateness for HTA is provided in Table 3. We provide an overview of methods in each group in the following subsections. We do not further describe the ITT analysis, since it does not attempt to adjust for nonadherence.

**Table 2:** Estimands, Causal Interpretation of Estimates, and Key Assumptions for Nonadherence Adjustment Methods.

Method	Estimand^a^	Estimand Attribues	Causal Interpretation of the Estimate	Key Assumptions
ITT	The effect of treatment assignment (not the effect of treatment itself)	Entire study population; ignoring events such as nonadherence and dropout	The average causal effect of treatment assignment on the survival outcome in a particular study (regardless of adherence, dropout, etc.)	The randomization assumption (i.e., group membership is randomly assigned), which implies that groups are comparable or exchangeable
PP	The effect of following the study protocol	Subpopulation of the protocol compliers in the study; excluding protocol noncompliers from the analysis set	The average causal effect of treatment on the survival outcome in individuals who adhered to the protocol in terms of eligibility, adherence, outcome assessment, etc.	The groups of patients who adhered to the protocol in each arm are comparable after covariate adjustment.
AT	The effect of treatment actually received	Subpopulation of patients who initiated treatment, with patients who switched treatment analyzed with the group they switched to regardless of randomization	The average causal effect of treatment on the survival outcome among individuals who actually received the treatment in the experimental group (including control group patients who switched onto the experimental treatment) compared to those who actually received the standard treatment (or those who actually did not receive the treatment in placebo-controlled trials) regardless of treatment assignment	The group of patients who received the treatment is comparable to those who did not, regardless of their treatment assignment.
CPH with PLE	CACE	Subpopulation who adhered to the protocol, excluding patients who did not adhere to the protocol in each arm of the study	The average treatment effect on the survival outcome in the complier subpopulation (patients who adhered to the protocol)	Covariates included in the model are independent of adherence.
MCC	CACE	As above	As above	The Markov assumption Time-varying adherence depends on the history of adherence Latent and ignorable missing data mechanism
Wtd PP	CACE	As above	As above	Patient population consists of 3 (possibly latent) subgroups: “ambivalent,”“insisters,” and “refusers”
C-PROPHET	CACE	As above	As above	The exclusion restriction assumption
IV	CACE	As above	As above	The exclusion restriction assumption Randomization has no effect on the probability of adherence to treatment Monotonicity assumption
MSMs with IPCW/IPTW	The effect of treatment had everyone remained adherent to the protocol	Entire study population; had everybody adhered to the protocol with perfect adherence to the prescribed dosing regimen or had everybody adhered to the protocol at an alternative level of adherence to the prescribed dosing regimen than what was observed in the trial (e.g., real-world adherence level)	The average causal effect of treatment that would have been observed if everybody adhered to the protocol. MSMs estimate the average treatment effect in the entire population, but the causal effect in a subset of the population (defined by a combination of variables L) can also be estimated. The IPCW estimand can also be interpreted as a comparison of the potential (counterfactual) outcomes under different levels of adherence in the same group of subjects.	No unmeasured confounders Positivity assumption
SNFTMs with G-estimation	The effect of treatment had everyone remained adherent to the protocol	As above	The average treatment effect that would have been observed if everybody adhered to the protocol (or remained at a particular adherence level such as real-world adherence level). SNFTMs can be used to estimate the average causal effect in a subset of the population defined by a combination of factors (L), e.g., men, patients aged >60 years	No unmeasured confounders Survival times and treatment-free survival times are proportional by an unknown factor that depends on the exposure.
RPSFTMs with G-estimation	The effect of treatment had everyone remained adherent to the protocol	As above	The average treatment effect that would have been observed if everybody adhered to the protocol compared to none treated.	The randomization assumption The common treatment effect assumption Survival times and treatment-free survival times are proportional by an unknown factor that depends on the exposure.
PKPD method	The effect of following a particular adherence pattern in the study population	Entire study population; given a particular pattern of adherence to the prescribed dosing regimen	The average causal effect of treatment if individuals followed a particular adherence pattern	The exclusion restriction assumption Correctly specified model

AT, as treated; CACE, complier average causal effect; CPH, Cox proportional hazards; C-PROPHET, Complier PROPortional Hazards Effect of Treatment; IPCW, inverse probability of censoring weighting; IPTW, inverse probability of treatment weighting; ITT, intention to treat; IV, instrumental variable; MCC, Markov compliance class; MSMs, marginal structural models; PKPD, pharmacokinetics and pharmacodynamics; PLE, partial likelihood estimator; PP, per protocol; RPSFTMs, rank-preserving structural failure time models; SNFTMs, structural nested failure time models; Wtd PP, weighted per protocol.

a. The estimand is the parameter of interest defined using 4 attributes: 1) the population, 2) the outcome variable or endpoint, 3) the specification of how to deal with intercurrent events (e.g., include compliers only), and 4) the population-level summary of the outcome variable. The description of the estimand in this table is focused on 2 attributes (the population and specification of how to deal with intercurrent events), as the other 2 attributes (the outcome variable and the population-level summary of the outcome variable) are expected to be similar in the context of time-to-event outcomes.

**Table 3:** Appropriateness of Estimand for the HTA Context, Types of Nonadherence, Possibility to Account for Real-World Adherence Levels, and Suitability of the Effectiveness Estimates for HTA Using the Alternative Adjustment Methods

Method	Appropriateness of Estimand for the HTA Context^a^	Type of Nonadherence That Can Be Adjusted for Using the Method		Possibility to Account for Real-World Nonadherence Levels^c^	Suitability of the Method for Use in HTA	Notes
		Initiation, Implementation, Persistence	Random, Explainable Nonrandom, No-Random^b^			
ITT	Yes	None	None	No	No	The estimand is marginalized to the entire population. Cannot estimate counterfactual estimands (i.e., treatment effectiveness given adherence levels in the real world).
PP	No	Initiation, implementation, persistence	Random	No	No	The estimand is not marginalized to the entire population. Excluding the protocol noncompliers may break the randomization balance, leading to selection bias if protocol noncompliance is related to underlying prognosis.
AT	No	Initiation	Random	No	No	Does not respect the randomization balance, which may lead to selection bias. Cannot estimate counterfactual estimands.
CPH with PLE	No	Initiation	Random, explainable nonrandom	No	No	The CACE estimand used by all 5 methods is not marginalized to the entire population. The compliers class is a latent group of patients that is not identifiable at baseline, making it difficult for policymakers to make resource allocation decisions based on CACE estimand. IV can estimate effectiveness given real-world adherence level based on the counterfactual outcome framework.
MCC	No	Initiation, implementation	Random	No	No	
Wtd PP	No	Initiation	Explainable nonrandom	No	No	
C-PROPHET	No	Initiation	Nonrandom	No	No	
IV	No	Initiation, implementation, persistence	Nonrandom	Yes	No	
MSMs	Yes	Initiation, implementation, persistence	Explainable nonrandom	Yes	Yes	Effectiveness estimates are marginalized to entire study population. Can be used to account for real-world adherence levels. RPSFTM only estimates the “all treated” v. “nontreated” estimand, making it applicable to adjust for “initiation” type of adherence only.
SNFTMs	Yes	Initiation, implementation, persistence	Explainable nonrandom	Yes	Yes	
RPSFTMs	Yes	Initiation	Nonrandom	Yes	Yes	
PKPD	Yes	Initiation, implementation, persistence	Explainable nonrandom	Yes	Yes	The estimand is marginalized to the entire population. Can estimate effectiveness given different adherence patterns.

AT, as treated; CACE, complier average causal effect; CPH, Cox proportional hazards; C-PROPHET, Complier PROPortional Hazards Effect of Treatment; HTA, health technology assessment; IPCW, inverse probability of censoring weighting; IPTW, inverse probability of treatment weighting; ITT, intention to treat; IV, instrumental variable; MCC, Markov compliance class; MSMs, marginal structural models; PKPD, pharmacokinetics and pharmacodynamics; PLE, partial likelihood estimator; PP, per protocol; RPSFTMs, rank-preserving structural failure time models; SNFTMs, structural nested failure time models; Wtd PP, weighted per protocol.

a. In the HTA context, the estimand of interest includes the entire study population, and this should be identifiable at baseline for resource allocation decision making.

b. This column specifies the type of nonadherence that each adjustment method is capable of dealing with in terms of random (nonselective) nonadherence, explainable nonrandom (selective) nonadherence (i.e., nonadherence explainable by observed covariates), or no-random (selective) nonadherence.

c. In the HTA context, methods for adjusting trial data for nonadherence need to be capable of reestimating treatment effectiveness for any given level of adherence (e.g., real-world adherence levels).

### Simple Methods

#### Per-protocol analysis

The standard per-protocol (PP) analysis strategy attempts to estimate the treatment effect among adherent patients by excluding protocol noncompliers.^45^ PP can deal with random (nonselective) types of nonadherence (initiation, implementation, persistence). The main concern is that excluding some patients from the analysis may undermine the prognostic balance generated by the randomization, which may introduce selection bias. This is likely to be the case if nonadherence is not random (i.e., if nonadherence is influenced by other patient characteristics and prognostic factors).^55^ Even if prognostic factors that are associated with nonadherence are correctly identified, PP analysis will introduce bias in the presence of time-dependent confounding.

#### As-treated analysis

The as-treated (AT) method attempts to adjust for the random initiation type of nonadherence. AT estimates the average causal effect (ACE) among patients who actually received the treatment compared to those who did not receive the treatment, assuming they are similar regardless of randomization.^34^ The main problem with this approach is that the group who actually received the treatment is unlikely to be comparable to the group who did not, making this approach prone to selection bias.^56^ AT analysis is less commonly used in practice compared with ITT and PP conventional methods.

### Principal Stratification Methods

#### Cox proportional hazards model with partial likelihood estimator

The Cox proportional hazards (CPH) model with partial likelihood estimator (PLE) is a method for estimating the treatment effect adjusted for initiation nonadherence at baseline while respecting the randomization.^41^ This is a semiparametric model whereby the treatment effect on the distributions of failure times is the parametric part. In the basic model, an individual with covariates (*k, z*_0_, *z*) will have a hazard function presented in equation (1).

(1) $$\exp\left(\gamma_{T}z_{0}+\beta z+\gamma_{k}\right)\lambda \left(t\right),$$

where $\gamma_{T}$ is the treatment effect in compliers (CACE estimand) expressed in terms of the hazard at time t for a cumulative hazard function Λ_*k*_(*t*) (this is only observable for the compliers class), $\gamma_{k}$ is the adherence class of the *k*th individual, $z_{0}$ is a vector of baseline covariates, and *z* is a set of time-dependent covariates. The standard method assumes that covariates are independent of nonadherence. The method can be used to adjust for nonadherence in situations where nonadherence is dependent on baseline covariates, but this approach requires a more complex estimator.^41^ The key limitation of this method is the difficulty of modeling time-varying treatments and other types of nonadherence beyond initiation.

#### Markov compliance class model in a 3-stage method

The Markov compliance class (MCC) model can accommodate both initiation and time-varying nonadherence (implementation) in the context of longitudinal studies where patients are randomized at baseline and randomization is maintained over time.^42^ The concept of this method is based on specifying 2 possible adherence classes that are applied at specified time points; for example, 5 time points results in a total of 32 (2^5^) adherence patterns. A stratification strategy can then be used to stratify adherence patterns into superclasses (low compliers, decreasing compliers, and high compliers). This can be used to estimate the CACE estimand among the compliers superclass. Model (2) can then be used to account for the relationship between adherence and survival time at time *t*.

(2) $$h\left(t|U_{i}=k\right)=h_{0}\left(t\right)\exp\left(\beta_{k}I\left(U_{i}=k\right)\right),$$

where $\beta_{k}$ for one of the adherence superclasses is assumed 0 for identification (reference superclass) and $U_{i}$ is individual *i*’s adherence superclass for a k number of superclasses.^42^ As a limitation, the method cannot deal with time-dependent confounding.

#### Weighted per-protocol analysis with expectation-maximization estimator

The weighted per-protocol (Wtd PP) method estimates the CACE by focusing on the ambivalent (compliers) class. The method attempts to deal with treatment initiation over time with 2 main features: 1) proposing a Wtd PP estimator by using time-varying weights that are subject specific (depend on baseline and time-dependent covariates) in a survival model and 2) proposing an expectation-maximization algorithm to maximize the full likelihood (FL) and PLEs.^47^ The method was developed to adjust for time-dependent confounders, which are associated with nonadherence. The partial likelihood estimator used by this model is similar to that used in the CPH with PLE approach (model (1)). Details of the FL estimator are reported in Li and Gray.^47^

#### Compliers PROPortional Hazards Effect of Treatment

The Compliers PROPortional Hazards Effect of Treatment (C-PROPHET) identifies adherent patients (initiation at baseline) and estimates the treatment effect in this group, adjusting for baseline covariates.^39^ C-PROPHET is a semiparametric model with the parametric side being the effect of the exposure on the survival times distribution.^39^ If individual patients who actually adhered to the protocol can be predicted at baseline in the intervention and control arms of an RCT, then one could fit a PH model for this study subpopulation to estimate the treatment effect.

The C-PROPHET model assumes that the hazard of survival time (T_*i*_) is as provided in equation (3).^39^

(3) $$\lambda \left(t|Z_{i}=1,E_{1i}=1\right)=\lambda \left(t|Z_{i}=0,E_{1i}=1\right)\exp\left(\psi_{0}\right),$$

where *Z_i_* is the randomization variable for individual *i* ($Z_{i}=1$ for the intervention group, $Z_{i}=0$ for the control group) and $E_{1i}$ represents the principal stratum at the treatment initiation stage. The parameter $\psi_{0}$ denotes the causal proportional hazards effect in the subpopulation of compliers. This is the parameter of interest that is called C-PROPHET.^39^ In terms of limitations, the method cannot be used to adjust for time-dependent nonadherence.

#### Instrumental variable method

The instrumental variable (IV) method can be used for adjusting for all types of nonadherence using a binary adherence variable. The method relies on the exclusion restriction assumption; that is, the IV affects the survival outcome only through its effects on the exposure. Three variants of the IV approach were identified: 1) IV with likelihood estimator,^32^ 2) IV with plug-in nonparametric empirical maximum likelihood estimator (PNEMLE),^43^ and 3) transformation promotion time cure model with maximum likelihood estimator (MLE).^48^

The IV with likelihood estimator works by classifying individuals in the trial population into 4 groups (similar to the classification used by MCC method). The estimator should be used to calculate the probability of having the case-specific event of interest at time *t* for each latent adherence class. Treatment effect in terms of hazard ratio (HR) can then be computed. This method was further applied to estimate adherence-adjusted cost-effectiveness using RCT data.^32^

The PNEMLE approach assumes the following survival functions for compliers in the intervention group (equation (4)), denoted as *S_c_*_1_(*V*), and control group (equation (5)), denoted as *S_c_*_0_(*V*), while never-takers have similar survival function in both groups, denoted as *S_nt_*(*V*).

(4) $$S_{T}|R=1\left(V\right)=\pi_{c}S_{c1}\left(V\right)+\left(1-\pi_{c}\right)S_{\mathrm{nt}}\left(V\right),$$

(5) $$S_{T}|R=0\left(V\right)=\pi_{c}S_{c0}\left(V\right)+\left(1-\pi_{c}\right)S_{\mathrm{nt}}\left(V\right),$$

where π_*c*_ is the fraction of compliers in the intervention group.

The IV extension using transformation promotion time cure model is a semiparametric model for estimating CACE and complier effect on survival probability (CESP) estimands. Further details of this extension are reported in Gao and Zheng.^48^ By using an IV approach, the analyst can deal with time-dependent confounding. The main drawback of this method is finding an instrumental variable that meets all the criteria of a valid IV^15^; an inadequate IV can lead to an imprecise and/or biased estimate.

### G-Methods

#### Marginal structural models with inverse probability of censoring weighting/inverse probability of treatment weighting

This method can be used to adjust for all types of nonadherence by censoring individuals at the first time they become nonadherent and then use inverse probability of censoring weighting (IPCW) for estimating the ACE of treatment using marginal structural models (MSMs).^22^ The IPCW can be used to obtain a valid treatment effect by adjusting for baseline and time-dependent confounders. IPCW makes the “no unmeasured confounding” assumption, that is, the assumption of explainable nonrandom nonadherence by measured time-dependent confounders.^13,22^ Stabilized weights are used because unstabilized weights can be inefficient. In practice, the analyst should construct stabilized weights (${\hat{w}}_{\mathrm{it}}^{\mathrm{stab}})$ for each individual *i* in time interval *t* by multiplying all the probabilities of remaining uncensored (adherent) up to time *t* using equation (6).

(6) $${\hat{w}}_{\mathrm{it}}^{\mathrm{stab}}=\overset{t}{\underset{k=0}{\Pi }}\frac{1}{1-{\hat{p}}_{\mathrm{ik}}}/\overset{t}{\underset{k=0}{\Pi }}\frac{1}{1-{\hat{p}}_{0\mathrm{ik}}}=\overset{t}{\underset{k=0}{\Pi }}\frac{1-{\hat{p}}_{0\mathrm{ik}}}{1-{\hat{p}}_{\mathrm{ik}}},$$

where ${\hat{p}}_{\mathrm{ik}}$ is the predicted probability of nonadherence in time interval *k* given the randomization group and adjusting for baseline and time-dependent covariates, and ${\hat{p}}_{0\mathrm{ik}}$ is the probability of nonadherence given the randomization group and adjusting for baseline covariates only. A pseudo-population should be created using the IPCW, and then any survival analysis (e.g., a Cox partial likelihood estimator) can be applied for estimating adherence-adjusted effectiveness. The main limitation of IPCW is the assumption of no unmeasured confounders, which cannot be proven empirically.

As an alternative approach to IPCW, one could allow individuals to become adherent again following a period of nonadherence—this can be modeled using the inverse probability of treatment weighting (IPTW) approach.^35^ The key feature of this method is that it allows for modeling longitudinal adherence patterns where patients follow erratic adherence behaviors in implementing the prescribed dosing regimen (i.e., on/off adherence patterns).

#### Structural nested failure time models with G-estimation

The structural nested failure time models (SNFTMs) can be applied to adjust for all types of nonadherence by controlling for time-dependent confounding using the G-estimation technique.^31^ The model relates the individual’s observed survival time and treatment history to the counterfactual outcome. In the SNFTM framework, the no unmeasured confounding assumption implies that the potential outcome does not add to the prediction model for treatment initiation, conditional on other covariates included in the model. To formally explain the G-estimation procedure, let us assume the treatment effect model in equation (7).^57^ We fit a logistic regression model to obtain the coefficients in equation (8).

(7) $$Y^{t}\sim X_{\psi }\left(t\right)\mathrm{given}\left({\bar{A}}_{t},{\bar{L}}_{t}\right),$$

(8) $$P\left[A\left(t\right)\right]=\beta_{0}\left(t\right)+\beta_{1}A\left(t-1\right)+\beta_{2}L\left(t\right)+\beta_{3}X_{\psi },$$

where $Y^{t}$ is the observed survival time, ~means has the same distribution as, $X_{\psi }\left(t\right)$ is the counterfactual outcome, ${\bar{A}}_{t}$ is the past treatment, ${\bar{L}}_{t}$ is the history of covariates, and P[A(t)] is the probability of initiating the treatment at time *t*.

G-estimation is used to search for ψ value, which adds the least to the prediction model (i.e., treatment initiation is independent of counterfactual outcomes). This means we search for a value of $\hat{\psi }$ that results in a $X_{\psi }$ term having a coefficient $\beta_{3}=$ 0 in model (8). That value of ψ provides the best estimates of counterfactual survival times adjusted for nonadherence. The main limitation of SNFTMs is the potential biases related to the no unmeasured confounding assumption, which cannot be formally tested.

#### Rank-preserving structural failure time models with G-estimation

The rank-preserving structural failure time model (RPSFTM) is a semiparametric model for adjusting for initiation nonadherence using the randomization factor, observed survival time, and treatment history.^36^ The method relies on the “common treatment effect” assumption (equal treatment effect regardless of when the treatment was initiated but relative to the time for which the treatment was received). It also relies upon the randomization of the trial, meaning that counterfactual survival times are equal between groups.

A simple RPSFTM (equation (9)) can be constructed to estimate the counterfactual survival time ($T_{i}^{0}$).^14,38^

(9) $$T_{i}^{0}=\int_{0}^{T_{i}}\exp[-\psi Z_{i}A_{i}(t)]\mathrm{dt},$$

where $Z_{i}$ is the randomization variable, $A_{i}$ is a binary adherence variable that equals 1 when a patient initiated the treatment and 0 otherwise, $T_{i}$ is the observed survival time, and the factor exp(ψ) is the causal effect (the value by which survival time is shrunk or expanded as an effect of the treatment). At the “true” value of the parameter ψ (which we can find using G-estimation), the counterfactual survival between randomized groups will be equal, and that value of ψ would be the point estimate of the treatment effect.

RPSFTM allows us to deal with time-dependent initiation issues and can deal with time-dependent confounding. The original RPSFTM was extended to incorporate baseline covariates to improve the precision of estimators^37,38^ and uses recensoring to allow the method to deal with potentially informative censoring in the counterfactual data set.^33,40^ As limitations, the RPSFTM can only be used for adjusting for the initiation type of nonadherence, and it relies on the common treatment effect assumption, which is difficult to test.

### Pharmacometrics-Based Methods

#### Pharmacokinetics and pharmacodynamics–based method

The Pharmacokinetics and pharmacodynamics (PKPD)–based methods model all types of nonadherence for estimating treatment effectiveness. PKPD-based methods require model development and fitting using appropriate data, typically collected during each phase of clinical drug development, as well as simulation based on different patterns of adherence, dosing schedules, and patient characteristics where covariate effects are relevant. The pharmacodynamic endpoint may be of direct relevance (e.g., anticoagulant international normalized ratio [INR]) or may require extrapolation to estimate the link between the PKPD parameter and the outcome of interest (e.g., risk of cardiovascular events) using evidence from the literature.^44,58^ PKPD makes the exclusion restriction assumption, that is, randomization affects the outcome only through the exposure treatment.

The PKPD method has been extended for modeling varying nonadherence and estimating adherence-adjusted cost-effectiveness of treatments.^44,49^ The main limitation of this method is its reliance on an accurate model specification and PKPD data, which might not be routinely available in RCTs or observational studies across disease areas.

### Appropriateness of Nonadherence Adjustment Methods to the HTA Context

The results based on the criteria applied for assessing appropriateness (suitability of the estimand, type of nonadherence, and possibility to account for real-world nonadherence levels) for each of the identified adjustment methods is provided in Table 3. Five methods (ITT, MSMs, SNFTMs, RPSFTMs, and PKPD) generate the estimand that is appropriate for HTA (covering the entire study population), with only 3 of these being capable of accounting for all types of nonadherence (MSMs, SNFTMs, and PKPD). Five methods are thought to be capable of reestimating effectiveness for real-world levels of nonadherence. When looking across all 3 facets of estimating effectiveness for HTA, g-methods and PKPD appear to be more appropriate.

The main differences between the 4 classes of methods are the estimands, assumptions, and the types of nonadherence that each method is capable of dealing with. Simple methods are only valid in the presence of random (nonselective) nonadherence. Principal stratification methods are capable of adjusting for some types of nonadherence, but their estimands seem inappropriate for the HTA context based on the criteria we set out in the Methods section. Both g-methods and PKPD can deal with real-world nonadherence, and their estimands are appropriate for HTA. G-methods are similar in terms of their capability for adjusting effectiveness estimates for counterfactual nonadherence levels. However, PKPD is a unique method that uses a different approach compared to g-methods.

In practice, the analyst could apply g-methods to individual patient-level data from an RCT to reestimate treatment effectiveness (adjusted for nonadherence) for populating cost-effectiveness models. Real-world adherence levels could be estimated from registry data or observational studies. All g-methods could be applied using standard software (e.g., SAS, Stata, or R).^12,59–61^ While g-methods could be applied to real RCT data sets, the PKPD approach relies on simulating an RCT data set based on a specified pattern of nonadherence (e.g., real-world adherence) and then uses the simulated data for generating the adjusted estimates. This would require data (including PKPD data) collected at different phases of clinical drug development. The PKPD method can be applied using a specialist software (e.g., NONMEM) or standard software (e.g., R) for simulating the data set.^62^

## Discussion

A total of 12 methods for adjusting for nonadherence in the context of time-to-event outcomes were identified and briefly described in this article. The proposed taxonomy classifies adjustment methods into 4 groups: 1) simple methods, 2) principal stratification methods, 3) g-methods, and 4) pharmacometrics-based methods. Each method makes specific assumptions and has associated limitations, and many of these assumptions are nontestable. Identification and collection of baseline and time-dependent confounders were identified as crucial for adjusting for nonadherence.

The purpose of adjustment was highlighted as a fundamentally important issue as estimands differ between the methods, as do the practicalities of using the method to reestimate effectiveness for alternative levels of adherence. G-methods and PKPD appear more appropriate for adjusting effectiveness estimates given real-world adherence levels and the likely existence of time-dependent confounding in RCT data sets. Simple methods and principal stratification methods cannot reestimate effectiveness based on alternative, suboptimal adherence levels. The Wtd PP method uses weights similar to IPCW, but the estimand is restricted to the complier subpopulation.

Many potentially relevant papers with a focus on cost-effectiveness aspects were excluded as these did not provide a methodological contribution. This gap in the methodological literature on CEA for modeling the link between nonadherence and treatment effectiveness is consistent with findings from other studies.^8,63^ A previous review by Hughes et al.^27^ reported 5 methods for adjusting cost-effectiveness for nonadherence, which was focused on pharmacoeconomic models rather than the impact of nonadherence on effectiveness. In that review, the PKPD approach is the only method relevant to our review, with the other methods being health-economic models (decision tree, Markov, discrete-event simulation) for incorporating adherence-adjusted treatment effects in economic evaluations.

Many of the methods identified by our review have been described and compared (mostly in pairwise comparisons) in the methodological literature.^13,14,34,64,65^ Mostazir et al.^66^ published a review of methods for handling nonadherence to intervention protocols in RCTs that identified some of the methods; however, their review missed several relevant methods due to the restricted search strategy used. The limitations of simple methods in adjusting for nonadherence are consistently reported in the methodological literature.^33,34,39,45,56,64,67^ It has been noted previously that principal stratification methods require a binary adherence variable (e.g., compliers/noncompliers), which may be problematic as a threshold is required, and this is often arbitrarily decided (e.g., 80% adherence level).^68^ This may also be an issue for g-methods and PKPD methods, where, in adjusting for nonadherence, we first need to define what constitutes “adherence.” This review has identified which nonadherence adjustment methods are likely to be useful in an HTA context. However, the remaining methods all have limitations, and their performance in relevant scenarios is unknown.

This review has used novel iterative search techniques and followed international guidelines^16–18^ but has limitations. First, a higher number of papers were excluded at the title screening stage because the paper’s title was not relevant. Second, we excluded non-peer-reviewed reports and other gray literature. While these two limitations might be an issue, the final list of included papers was checked by 2 experts, and we are confident that no important relevant method was missed. Third, minor variants of methods extensions are not included (e.g., proposing alternative censoring mechanisms for IPCW),^69^ which is inevitably a subjective decision. These decisions were based on discussions among the authors. Finally, the review does not assess the performance of the alternative methods; therefore, further research (well-conducted simulation studies) is warranted to provide recommendations for application in the HTA context.

In conclusion, economic evaluations frequently ignore the adjustment of treatment effectiveness for patient nonadherence, which carries the risk of producing misleading cost-effectiveness evidence if adherence levels in the real world differ from trials. A range of statistical methods is available for adjusting estimates of treatment effectiveness in the presence of patient nonadherence, although g-methods and PKPD appear to be more promising to account for real-world adherence levels in HTA. Further research is warranted to assess the performance of these methods.

## Supplemental Material

Appendix_A_online_supp – Supplemental material for Statistical Methods for Adjusting Estimates of Treatment Effectiveness for Patient Nonadherence in the Context of Time-to-Event Outcomes and Health Technology Assessment: A Systematic Review of Methodological Papers Click here for additional data file. Supplemental material, Appendix_A_online_supp for Statistical Methods for Adjusting Estimates of Treatment Effectiveness for Patient Nonadherence in the Context of Time-to-Event Outcomes and Health Technology Assessment: A Systematic Review of Methodological Papers by Abualbishr Alshreef, Nicholas Latimer, Paul Tappenden, Ruth Wong, Dyfrig Hughes, James Fotheringham and Simon Dixon in Medical Decision Making

## Supplemental Material

Appendix_B_online_supp – Supplemental material for Statistical Methods for Adjusting Estimates of Treatment Effectiveness for Patient Nonadherence in the Context of Time-to-Event Outcomes and Health Technology Assessment: A Systematic Review of Methodological Papers Click here for additional data file. Supplemental material, Appendix_B_online_supp for Statistical Methods for Adjusting Estimates of Treatment Effectiveness for Patient Nonadherence in the Context of Time-to-Event Outcomes and Health Technology Assessment: A Systematic Review of Methodological Papers by Abualbishr Alshreef, Nicholas Latimer, Paul Tappenden, Ruth Wong, Dyfrig Hughes, James Fotheringham and Simon Dixon in Medical Decision Making
