# Supplementary material for: Statistical Methods for Adjusting Estimates of Treatment Effectiveness for Patient Nonadherence in the Context of Time-to-Event Outcomes and Health Technology Assessment: A Systematic Review of Methodological Papers
Source: Med Decis Making. 2019 Oct 24;39(8):910–25. doi: 10.1177/0272989X19881654 (PMC6900590; doi:10.1177/0272989X19881654)
Supplement: Appendix_A_online_supp – Supplemental material for Statistical Methods for Adjusting Estimates of Treatment Effectiveness for Patient Nonadherence in the Context of Time-to-Event Outcomes and Health Technology Assessment: A Systematic Review of Methodological Papers [file Appendix_A_online_supp.pdf]

Appendix A: Search terms, strategies and results

A1: Search terms

Patient adherence terms include compliance, adherence, pharmacoadherence, persistence, persistency, concordance, initiation, implementation, noncompliance, nonadherence, nonpersistence, discontinuation, pharmionics, therapeutic alliance, patient irregularity or treatment refusal. MeSH headings for methods include "models, statistical" or "models, structural" or "models, economic" or "models, econometric" or "models, biological" or "survival analysis" or "logistic models" or "proportional hazards models".

A2: First iteration searches and results

MEDLINE(R) Epub Ahead of Print, In-Process & Other Non-Indexed Citations, MEDLINE(R) Daily, MEDLINE and Versions(R): Ovid 1946 to February 9 2018

#	Terms	Results
1	(compliance or adherence or pharmacoadherence or persistence or persistency or concordance or initiation or implementation or noncompliance or nonadherence or nonpersistence or discontinuation or pharmionics or therapeutic alliance or patient irregularity or treatment refusal).ti.	120596
2	*Models, Structural/	2122
3	*models, statistical/	28208
4	*models, economic/ or *models, econometric/	4387
5	1 and (2 or 3 or 4)	245

MEDLINE(R) Epub Ahead of Print, In-Process & Other Non-Indexed Citations, MEDLINE(R) Daily, MEDLINE and Versions(R): Ovid 1946 to March 28 2018 – Adding "Models, Biological" MeSH heading.

3rd April 2018

1	(compliance or adherence or pharmacoadherence or persistence or persistency or concordance or initiation or implementation or noncompliance or nonadherence or nonpersistence or discontinuation or pharmionics or therapeutic alliance or patient irregularity or treatment refusal).ti.	121991
2	*Models, Structural/	2128
3	*Models, Statistical/	28482
4	*models, economic/ or *models, econometric/	4416
5	*Models, Biological/	97476
6	1 and (2 or 3 or 4)	249
7	1 and 5	625
8	limit 7 to humans	324
9	8 not 6	316

Embase: Ovid 1974 to 2018 March 30
3rd April 2018

1	(compliance or adherence or pharmacoadherence or persistence or persistency or concordance or initiation or implementation or noncompliance or nonadherence or nonpersistence or discontinuation or pharmionics or therapeutic alliance or patient irregularity or treatment refusal).ti.	159985

2	*structural model/	153
3	*statistical model/	21073
4	*economic model/	491
5	*biological model/	56706
6	1 and (2 or 3 or 4)	143
7	1 and 5	381
8	limit 7 to human	156
9	6 or 8	298

Cochrane Library: Wiley
3rd April 2018

#1	(compliance or adherence or pharmacoadherence or persistence or persistency or concordance or initiation or implementation or noncompliance or nonadherence or nonpersistence or discontinuation or pharmionics or therapeutic alliance or patient irregularity or treatment refusal):ti (Word variations have been searched)	12301
#2	MeSH descriptor: [Models, Structural] this term only	25
#3	MeSH descriptor: [Models, Statistical] this term only	1577
#4	MeSH descriptor: [Models, Economic] this term only	1578
#5	MeSH descriptor: [Models, Econometric] this term only	470
#6	MeSH descriptor: [Models, Biological] this term only	2370
#7	#1 and (#2 or #3 or #4 or #5)	52
#8	#1 and #6	16
#9	#7 or #8	67

Econlit: Ovid 1886 to May 3, 2018
8th May 2018

1	(compliance or adherence or pharmacoadherence or persistence or persistency or concordance or initiation or implementation or noncompliance or nonadherence or nonpersistence or discontinuation or pharmionics or therapeutic alliance or patient irregularity or treatment refusal).ti.	8632
2	model*.ti.	90932
3	(structural or statistical or economic or econometric or biological).ti.	114247
4	1 and 2 and 3	31

Web of Science: Clarivate Analytics
8th May 2018

# 1	TITLE: ((compliance or adherence or pharmacoadherence or persistence or persistency or concordance or initiation or implementation or noncompliance or nonadherence or nonpersistence or discontinuation or pharmionics or therapeutic alliance or patient irregularity or treatment refusal))	256,870
# 2	TITLE: (model*)	1,892,481
# 3	TITLE: ((structural or statistical or economic or econometric or biological))	706,793
# 4	#3 AND #2 AND #1	240

Scopus: Elsevier
8th May 2018

#1	(TITLE ((compliance OR adherence OR pharmacoadherence OR persistence OR persistency OR concordance OR initiation OR implementation OR noncompliance)) OR TITLE ((nonadherence OR	321,353

	nonpersistence OR discontinuation OR pharmionics OR therapeutic AND alliance OR patient AND irregularity OR treatment AND refusal)))	
#2	TITLE (model*)	2,338,498
#3	TITLE ((structural OR statistical OR economic OR econometric OR biologi cal))	905,167
#4	((TITLE ((compliance OR adherence OR pharmacoadherence OR persistence OR persistency OR concordance OR initiation OR implementation OR noncompliance)) OR TITLE ((nonadherence OR nonpersistence OR discontinuation OR pharmionics OR therapeutic AND alliance OR patient AND irregularity OR treatment AND refusal)))) AND (TITLE (model*)) AND (TITLE ((structural OR statistical OR economic OR econometric OR biological)))	323

A3: Second iteration searches and results

MEDLINE(R) Epub Ahead of Print, In-Process & Other Non-Indexed Citations, MEDLINE(R) Daily, MEDLINE and Versions(R): Ovid 1946 to May 16, 2018

22nd May 2018

#	Terms	Results
1	(compliance or adherence or pharmacoadherence or persistence or persistency or concordance or initiation or implementation or noncompliance or nonadherence or nonpersistence or discontinuation or pharmionics or therapeutic alliance or patient irregularity or treatment refusal).ti.	123742
2	*Models, Structural/	2129
3	*Models, Statistical/	28851
4	*models, economic/ or *models, econometric/	4476
5	*Models, Biological/	98419
6	1 and (2 or 3 or 4)	250
7	1 and 5	634
8	limit 7 to humans	329
9	8 not 6	321
10	*Survival Analysis/	2666
11	*Proportional Hazards Models/	1811
12	*Linear Models/	2498
13	*Logistic Models/	1675
14	Biometry/mt [Methods]	4244
15	Randomized Controlled Trials as Topic/sn [Statistics & Numerical Data]	4789
16	Cost-Benefit Analysis/sn [Statistics & Numerical Data]	981
17	Economics, Pharmaceutical/sn [Statistics & Numerical Data]	144
18	or/10-17	17625
19	pharmacometric*.tw.	388
20	causal inference.tw.	1455
21	proportional hazards.ti.	411
22	structural model*.ti.	1624
23	proportional hazards model*.ab.	20752
24	structural nested model*.ab.	27
25	marginal structural model*.ab.	518
26	structural proportional hazards.ab.	3
27	structural accelerated failure.ab.	7
28	compliance class model*.ab.	2
29	preserving structural failure.ab.	31
30	rank preserving structural.ab.	31
31	accelerated failure time.ab.	479
32	or/19-31	25187

33	18 or 32	41976
34	1 and 33	616
35	limit 34 to humans	523
36	35 not 9	520

Embase: Ovid 1974 to 2018 May 21
22nd May 2018

1	(compliance or adherence or pharmacoadherence or persistence or persistency or concordance or initiation or implementation or noncompliance or nonadherence or nonpersistence or discontinuation or pharmionics or therapeutic alliance or patient irregularity or treatment refusal).ti.	162336
2	*structural model/	160
3	*statistical model/	21307
4	*economic model/	506
5	*biological model/	57036
6	1 and (2 or 3 or 4)	146
7	1 and 5	385
8	limit 7 to human	157
9	6 or 8	302
10	*survival analysis/	645
11	*proportional hazards model/	1385
12	10 or 11	2017
13	pharmacometric*.tw.	568
14	causal inference.tw.	1544
15	proportional hazards.ti.	418
16	structural model*.ti.	1691
17	proportional hazards model*.ab.	32565
18	structural nested model*.ab.	26
19	marginal structural model*.ab.	681
20	structural proportional hazards.ab.	3
21	structural accelerated failure.ab.	9
22	compliance class model*.ab.	2
23	preserving structural failure.ab.	98
24	rank preserving structural.ab.	98
25	accelerated failure time.ab.	552
26	or/13-25	37594
27	1 and (12 or 26)	702
28	27 not 9	691

Web of Science: Clarivate Analytics
22nd May 2018

# 1	TITLE: ((compliance or adherence or pharmacoadherence or persistence or persistency or concordance or initiation or implementation or noncompliance or nonadherence or nonpersistence or discontinuation or pharmionics or therapeutic alliance or patient irregularity or treatment refusal))	257,485
# 2	TITLE: (model*)	1,896,217
# 3	TITLE: ((structural or statistical or economic or econometric or biological))	708,343
# 4	#3 AND #2 AND #1	240
# 5	TI=("survival analysis")	3,080
# 6	TI=("proportional hazards model*")	513
# 7	TI=("linear model*")	6,683
# 8	TI=("logistic model*")	789
# 9	TOPIC: (pharmacometric*)	464

# 10	TS=("causal inference")	3,246
# 11	TI=("proportional hazards")	823
# 12	TI=("structural model*")	4,117
# 13	TI=("proportional hazards model*")	513
# 14	TOPIC: ("proportional hazards model*")	20,669
# 15	TOPIC: ("structural nested model*")	43
# 16	TOPIC: (“marginal structural model*”)	1,192
# 17	TOPIC: (“structural proportional hazards”)	3
# 18	TOPIC: ("structural accelerated failure")	9
# 19	TOPIC: ("compliance class model*")	4
# 20	TOPIC: ("preserving structural failure")	37
# 21	TOPIC: ("rank preserving structural")	38
# 22	TOPIC: ("accelerated failure time")	849
# 23	#22 OR #21 OR #20 OR #19 OR #18 OR #17 OR #16 OR #15 OR #14 OR #13 OR #12 OR #11 OR #10 OR #9 OR #8 OR #7 OR #6 OR #5	40,195
# 24	#23 AND #1	575
# 25	#24 not #4	519

MathSciNet: American Mathematical Society
23rd May 2018
27 records

1.	"Title=(compliance or adherence or pharmacoadherence or persistence or persistency or concordance or initiation or implementation)"	7806	Compliance in title
2.	"Title=(noncompliance or nonadherence or nonpersistence or discontinuation or pharmionics)"	91	
3.	'Title=("therapeutic alliance" or "patient irregularity" or "treatment refusal")'	0	
4.	"Title=(model*)"	21005	Model in title
5.	"Title=(structural or statistical or economic or econometric or biological)"	36577	
6.	“Review Text=(survival analysis or proportional hazards model* or linear model* or logistic model*)"	507	Second iteration model terms
7.	"Review Text=(pharmacometric* or “causal inference“)"	439	
8.	"Review Text=(proportional hazards or structural model* or proportional hazards model* or structural nested model*)"	22	
9.	'Review Text=(marginal structural model* or structural proportional hazards or structural accelerated failure or compliance class model*)'	0	
10.	"Review Text=(preserving structural failure or rank preserving structural or accelerated failure time)"	5	
11.	"Title=(compliance or adherence or pharmacoadherence or persistence or persistency or concordance or initiation or implementation) AND Title=(model*) AND Title=(structural or statistical or economic or econometric or biological)"	16	1st search
12.	"Title=(noncompliance or nonadherence or nonpersistence or discontinuation or pharmionics) AND Title=(model*) AND Title=(structural or statistical or economic or econometric or biological)"	3	
13.	Title=(compliance or adherence or pharmacoadherence or persistence or persistency or concordance or initiation or implementation) AND Review Text=(survival analysis or proportional hazards model* or linear model* or logistic model*)" '	1	2nd search (a)
14.	'Title=(noncompliance or nonadherence or nonpersistence or discontinuation or pharmionics) AND Review Text=(survival analysis or proportional hazards model* or linear model* or logistic model*)'	0	

15.	"Title=(compliance or adherence or pharmacoadherence or persistence or persistency or concordance or initiation or implementation) AND Review Text=(pharmacometric* or “causal inference“)"	3	2nd search (b)
16.	'Title=(compliance or adherence or pharmacoadherence or persistence or persistency or concordance or initiation or implementation) AND Review Text=(proportional hazards or structural model* or proportional hazards model* or structural nested model*)'	0	
17.	'Title=(compliance or adherence or pharmacoadherence or persistence or persistency or concordance or initiation or implementation) AND Review Text=(preserving structural failure or rank preserving structural or accelerated failure time)'	0	
18.	"Title=(noncompliance or nonadherence or nonpersistence or discontinuation or pharmionics) AND Review Text=(pharmacometric* or “causal inference“)"	3	
19.	'Title=(noncompliance or nonadherence or nonpersistence or discontinuation or pharmionics) AND Review Text=(proportional hazards or structural model* or proportional hazards model* or structural nested model*)'	0	
20.	"Title=(noncompliance or nonadherence or nonpersistence or discontinuation or pharmionics) AND Review Text=(preserving structural failure or rank preserving structural or accelerated failure time)"	1	
21.	or/11-20	27	
