# Supplementary material for: Statistical Methods for Adjusting Estimates of Treatment Effectiveness for Patient Nonadherence in the Context of Time-to-Event Outcomes and Health Technology Assessment: A Systematic Review of Methodological Papers
Source: Med Decis Making. 2019 Oct 24;39(8):910–25. doi: 10.1177/0272989X19881654 (PMC6900590; doi:10.1177/0272989X19881654)
Supplement: Appendix_B_online_supp – Supplemental material for Statistical Methods for Adjusting Estimates of Treatment Effectiveness for Patient Nonadherence in the Context of Time-to-Event Outcomes and Health Technology Assessment: A Systematic Review of Methodological Papers [file Appendix_B_online_supp.pdf]

Appendix B: Supplementary Tables

Table S1 Inclusion and Exclusion Criteria Applied for Selection of Selection of Papers Included in the Review

Inclusion criteria	Exclusion criteria
Peer-reviewed methodological papers which describe the method(s) in detail such that they can be applied without the need for further assumptions;*	Non-peer reviewed reports, books or book chapters, theses, or other grey literature;
Methods explicitly applied to adjust for non-adherence in estimating treatment-effects for survival-time outcomes and/or cost-effectiveness;	Papers which merely apply previously developed method(s) without any additional extension to the original method(s)+;
Papers published from databases inception to date; and	Methods which are not explicitly applied to adjust for non-adherence to treatments
Papers published in the English language.	Methods based on aggregated data such as meta-analysis; or
	Theoretical papers with no application of the method.

* This criterion is not objective and required a judgement on my part informed by expert advice

+ This implied that the first paper proposing the method was included and any paper published afterwards with the application of the method without any methodological extension was excluded.

Table S2 Data Extraction Form

Basic information				Methodological origin			
Paper ID#	Author(s)	Year of publication	Journal	Method	Method acronym	Methods Group	Type of methodological contribution (original/extension)

Table S2 Data Extraction Form (continued)

Methodological origin (continued)			Theoretical suitability			
Was the method originally developed to adjust for non-adherence?	If not, what was the original context and how the method was adapted?	Does the method represent an extension to another method adjusting for non-adherence?	How does the method work?	What are the key assumptions?	What are the potential biases?	Why might the method not be appropriate?

Table S2 Data Extraction Form (continued)

Theoretical suitability (continued)		Application				
What are the advantages and disadvantages associated with the method?	What are the similarities and differences of the method compared to other methods identified?	Has the method been applied to adjust for non-adherence in a case/ simulation study?	What disease/ condition applied in the case/ simulation study?	What are intervention(s) assessed in the case/simulation study?	Outcome(s) assessed	What were the results compared to traditional approaches (ITT/PPT/AT)?

Table S3 Appraisal Framework

Domain	Issues considered
Origin of the method	Was the method originally developed to adjust for non-adherence?<br>If not, what was the original context and how the method was adapted?<br>Does the method represent an extension to another method adjusting for non-adherence?
Theoretical suitability	How does the method work?<br>What are the key assumptions?<br>What are the potential biases?<br>Why might the method not be appropriate?<br>What are the advantages and disadvantages associated with the method?<br>What are the similarities and differences of the method compared to other methods identified?
Application	Has the method been applied to adjust for non-adherence in a case study/simulation study?<br>What disease/condition is applied in the case/simulation study?<br>What is/are the intervention(s) assessed in the case/simulation study?<br>What were the results compared to simple methods (ITT/PP/AT), if compared?

Table S4 Characteristics of Papers Included in the Review (n=20)

Characteristic	No. (%)
**Year**	
2014-2018	6(30)
2009-2013	1(5)
2004-2008	3(15)
1999-2003	7(35)
Before 1999	3(15)
**Journal**	
Statistics in Medicine	6(30)
Biometrics	5(25)
Journal of the Royal Statistical Society	3(15)
Journal of the American Statistical Association	2(10)
Clinical Pharmacology & Therapeutics	1(5)
Communication in Statistics - Theory and Methods	1(5)
Epidemiology	1(5)
Value in Health	1(5)
**Type of study to which the method was applied**	
Simulation study	3(15)
Case study	6(30)
Both simulation study and case study	11(55)
**Disease area**	
Breast cancer	5(25)
AIDS	3(15)
Depression	2(10)
Lung cancer	2(10)
Leukaemia	2(10)
Colorectal cancer	1(5)
Atrial fibrillation	1(5)
Gout	1(5)
Vitamin A deficiency	1(5)
Hypertension	1(5)
Hypothetical condition	1(5)
